# Lymphocyte-to-Red Blood Cell Ratio—The Guide Star of Acute Coronary Syndrome Prognosis

**DOI:** 10.3390/healthcare12121205

**Published:** 2024-06-16

**Authors:** Cosmina Elena Jercălău, Cătălina Liliana Andrei, Lavinia Nicoleta Brezeanu, Roxana Oana Darabont, Suzana Guberna, Andreea Catană, Maria Diana Lungu, Octavian Ceban, Crina Julieta Sinescu

**Affiliations:** 1 Department of Cardiology, “Bagdasar Arseni” Emergency Hospital, University of Medicine and Pharmacy “Carol Davila”, 011241 Bucharest, Romania; roxana.darabont@umfcd.ro (R.O.D.); catanaandreea91@yahoo.com (A.C.); crina.sinescu@umfcd.ro (C.J.S.); 2 Department of Anaesthesia and Intensive Care, Fundeni Clinical Institute, 022328 Bucharest, Romania; laviniajipa11@yahoo.com; 3 Department of Cardiology, Emergency Hospital “Bagdasar-Arseni”, 050474 Bucharest, Romania; suzana.guberna@umfcd.ro (S.G.); maria-diana.lungu@rez.umfcd.ro (M.D.L.); 4 Economic Cybernetics and Informatics Department, The Bucharest University of Economic Studies, 010374 Bucharest, Romania; octavianceban1995@gmail.com

**Keywords:** myocardial infarction, atherosclerosis, prevention, prognosis, risk profile, inflammation

## Abstract

Background: Beneath the surface of the acute ST-elevation myocardial infarction (STEMI) iceberg lies a hidden peril, obscured by the well-known cardiovascular risk factors that tip the iceberg. Before delving into the potential time bomb these risk factors represent, it is crucial to recognize the obscured danger lurking under the surface. What secrets does the STEMI iceberg hold? To unveil these mysteries, a closer look at the pathophysiology of STEMI is imperative. Inflammation, the catalyst of the STEMI cascade, sets off a chain reaction within the cardiovascular system. Surprisingly, the intricate interplay between red blood cells (RBC) and lymphocytes remains largely unexplored in previous research. Materials and methods: The study encompassed 163 patients diagnosed with STEMI. Utilizing linear and logistic regression, the lymphocyte-to-red blood cell ratio (LRR) was scrutinized as a potential predictive biomarker. Results: There was a statistically significant correlation between LRR and the prognosis of STEMI patients. Building upon this discovery, an innovative scoring system was proposed that integrates LRR as a crucial parameter. Conclusions: Uncovering novel predictive markers for both immediate and delayed complications in STEMI is paramount. These markers have the potential to revolutionize treatment strategies by tailoring them to individual risk profiles, ultimately enhancing patient outcomes.

## 1. Introduction 

Atherosclerosis and inflammation are key factors in the development of coronary artery disease (CAD). The connection between inflammation and CAD is highlighted by the involvement of immune cells. These cells play a crucial role in orchestrating the inflammatory process, a central component in the onset and advancement of atherosclerosis [1].

Despite advancements in AMI patient care, high mortality and morbidity rates persist. Timely diagnosis, effective management, and accurate risk assessment are essential for enhancing patient outcomes [2].

In recent years, therapeutic strategies focusing on the molecular mechanisms involved in atherosclerosis and acute myocardial infarction have been increasingly developed. Before solely concentrating on new therapeutic approaches, it is valuable to revisit the pathophysiological mechanisms and the molecules involved in the process. In other words, we should focus forward but also regularly check the rearview mirror. Inflammation plays a significant role in all stages of atherosclerosis, from initiation to progression [3].

The inflammatory process resembles a domino puzzle: once it commences, the pieces are poised to topple and trigger a chain reaction. Following myocardial infarction, neutrophils are promptly recruited to the ischemic area, crucially initiating the inflammatory response. While neutrophils form part of the innate immune response, lymphocytes constitute the second group of cells to arrive at the site of myocardial injury, contributing to the adaptive immune response and bearing a crucial regulatory role [4,5]. An increasing number of studies demonstrate the correlation between elevated inflammatory markers, white blood cell counts, and the extent and severity of acute coronary syndrome.

Previous research has shown that the neutrophil-to-lymphocyte ratio (NLR) can be used as a predictor of disease severity and mortality in STEMI [6,7,8,9]. Moreover, considerable attention has been given to exploring the correlation between different types of leukocytes (monocyte/lymphocyte ratio—MLR, neutrophil/monocyte ratio—NMR) and patient outcomes in acute myocardial infarction [10,11,12]. But what about the relationship between red blood cells (RBC) and lymphocytes? Is there an interaction between these cells in the atherosclerosis process or myocardial infarction, and can this interaction influence prognosis? The LRR ratio (lymphocyte-to-red blood cells ratio) has only been analyzed in patients with neoplasia, specifically breast cancer [13].

Inspired by these findings, we sought to determine if this ratio could also predict the outcomes of STEMI patients during the acute in-hospital phase. Upon reviewing existing literature, we found that healthy red blood cells can have a beneficial impact on the immune system by promoting anti-inflammatory and anti-atherogenic mechanisms [14]. However, under conditions of oxidative stress, RBCs become oxidized or senescent, leading to changes in surface antigens and the release of extracellular vesicles. These vesicles, derived from altered RBCs, can enhance the proliferation and apoptosis of T cells and increase the production of pro-inflammatory and pro-atherogenic cytokines by T helper 1 cells. Additionally, oxidized RBCs are unable to effectively control the maturation of dendritic cells induced by lipopolysaccharides, resulting in the development of a pro-inflammatory Th1 cell response. It is crucial to note that this interaction between erythrocytes and lymphocytes initiates at the early stages of atherosclerosis. Previous literature suggests that the interplay between red blood cells and immune cells is a novel mechanism through which oxidative stress contributes to the progression of atherosclerotic disease [15,16]. This discovery paves the way for potential therapeutic interventions targeting the crosstalk between RBCs and immune cells. Hence, we aimed to investigate whether the interrelationship of RBCs and lymphocytes could be utilized to predict the prognosis of STEMI patients.

## 2. Materials and Methods

### 2.1. Study Design and Study Population

Patients admitted to the Cardiology Department of the “Bagdasar-Arseni” Emergency Hospital in Bucharest, Romania, with a primary diagnosis of STEMI between January 2022 and April 2023 were prospectively enrolled in our study. A final number of 163 STEMI patients were included and evaluated. Acute ST-elevation myocardial infarction was identified through the following: symptoms consistent with acute coronary syndrome (ACS), electrocardiograms showing STEMI, elevated cardiac biomarkers, and evident abnormal wall motion on echocardiography [17]. Thus, the inclusion criteria consisted of patients with a minimum age of 18 years old, bearing the diagnosis of STEMI, and with an early STEMI presentation (<12 h from symptom onset). The exclusion criteria were represented by (1) very late STEMI presentation >12 h; (2) incomplete medical record data; (3) patients with hematological or inflammatory autoimmune disease; (4) active infection; (5) liver or renal failure; (6) neoplasia; (7) patients who had recently undergone chemotherapy/radiotherapy; (8) patients with a history of surgery, trauma, blood transfusions in the past 30 days. After applying the exclusion criteria, 15 patients were not eligible for the study (2 suffered from neoplasia, 2 had multiple-organ failure, 2 had pneumonia, 2 had active tuberculosis, 1 had psoriasis, 1 had rheumatoid arthritis, 1 had thrombophilia, and 4 had very late STEMI presentation). Demographic data (age, sex), anthropometric measurements (body mass index—BMI), pathological history (prior myocardial infarction, other diseases which could interfere with the hematological parameters), cardiovascular risk factors (arterial hypertension, dyslipidemia, diabetes mellitus, obesity, smoking), laboratory data (NT-proBNP, admission and peak high-sensitive troponin, admission and peak CK/CK-MB, complete blood count, serum electrolytes, serum creatinine, serum glucose, ESR, fibrinogen, PSEP), as well as echocardiography findings (left ventricular systolic function) were recorded. We deemed the presence of an active infection to be a PSEP > 300 pg/mL and concurrent biological inflammatory syndrome. The lymphocyte-to-red blood cell ratio (LRR) was determined by dividing the absolute count of lymphocytes by the absolute count of red blood cells. Blood samples were obtained from each patient’s antecubital vein immediately upon admission.

Renal dysfunction was defined as an elevated serum creatinine level exceeding 1.3 mg/dL for men and 1.1 mg/dL for women. Concerning NT-proBNP, a marker of cardiac decompensation, an elevated level was characterized by values surpassing 300 pg/mL. Left ventricular ejection fraction (LVEF) values were acquired through transthoracic echocardiography conducted during hospitalization. The modified Simpson biplane method was utilized to calculate the left ventricular end-diastolic volumes (LVEDVs) and end-systolic volumes (LVESVs) from 4- and 2-chamber views, with LV volumes being adjusted for body surface areas. Regarding the definition, Killip class III refers to patients with STEMI and pulmonary edema, while Killip class IV defines patients with STEMI and cardiogenic shock (hypotension—a systolic blood pressure under 90 mmHg, and signs of low cardiac output) [17].

Demographic information and variables were used to determine TIMI risk score points. The total score ranged from 0 to 14 based on specific characteristics: age, presence of diabetes mellitus (DM) or hypertension (HT) or angina, heart rate below 100 bpm, systolic blood pressure below 100 mmHg, Killip class II–IV, weight under 67 kg, anterior myocardial infarction (MI) or left bundle branch block (LBBB) presentation, and latency exceeding 4 h. The TIMI risk score was calculated utilizing a computer program available at http://www.mdcalc.com/timi-risk-score-for-stemi/ (accessed on 20 April 2024) [18]. The Global Registry of Acute Coronary Events (GRACE) risk score points for in-hospital mortality, considering factors like age, creatinine levels, heart rate, systolic blood pressure, Killip class, cardiac arrest upon admission, elevated cardiac markers, and ST-segment deviation. The GRACE risk score was determined using a computer program found at https://www.mdcalc.com/calc/1099/grace-acs-risk-mortality-calculator (accessed on 20 April 2024) [19].

The HEART score was obtained by determining the history, ECG abnormalities, patients’ age, any risk factors, and tropoinin level using a computer program available at https://www.mdcalc.com/calc/1752/heart-score-major-cardiac-events (accessed on 20 April 2024) [20].

The outcomes were established as a composite of Killip class III/IV, the size of myocardial infarction, use of inotropes and vasopressors, kidney dysfunction, in-hospital mortality, and 30-day mortality. Patients were followed for 30 days after admission by consulting the national health authority’s online database, through which the survival/mortality rates were documented.

The research adhered to the guidelines set forth in the Declaration of Helsinki and received approval from the Ethics Committee at “Carol Davila” University of Medicine and Pharmacy in Bucharest, Romania (protocol code PO-35-F-03, issued on 1 October 2021). Written informed consent was obtained from all participants, and measures were implemented to guarantee the confidentiality of the data.

### 2.2. Statistical Analysis

In our study, receiver operating characteristics curves, distribution representations, and boxplots were utilized to display the results. The Python programming language, as well as traditional modeling tools such as NumPy, Pandas, and Statsmodels, were employed for data modeling. 

In assessing the statistical significance of the regression model, a *p*-value threshold of 0.05 was applied to ascertain the model’s statistical significance.

We employed linear regression analysis for continuous dependent variables such as CK, CK-MB, hsCTnI, NT-proBNP, LVEF, and creatinine. Logistic regression was used for binary outcome classification problems, including the use of inotropes and vasopressors, in-hospital mortality, 30-day mortality, TIMI, GRACE, and HEART scores. For linear regression, the model was represented as Y = $\beta_{0}+\beta_{1}X$, where Y represents the continuous dependent variable and *X*1 is the independent variable (LRR). The parameters and $\beta_{0}$ were estimated based on the sample data to interpret the relationship between X and Y. Significance testing of $\beta_{1}$ indicated the presence of a true relationship at the population level. Similarly, logistic regression yielded a model defined as Score for event Y = $\beta_{0}+\beta_{1}X$, where the score represents the probability of the event occurring (dependent binary variable Y), *X*1 is the LRR value (independent variable), and $\beta_{0}$ and $\beta_{1}$ are the estimated parameters. 

To express the results as probabilities, we have used the following equation: probability = 1/(1 + e^score^). 

## 3. Results

A total of 163 patients were enrolled in our study, and their baseline characteristics are summarized in Table 1. The mean age was 63 years old, and the ratio of male (63.9%) patients was higher than the female ratio (36.1%). All patients were of Caucasian ethnicity. The prevalence of cardiovascular risk factors was high: 79.4% were hypertensive, 36.1% had a BMI over 30 kg/m^2^, 39.2% had diabetes mellitus, 56.7% were dislipidemic, and 51.5% were smokers. The minimum systolic blood pressure was 90 mmHg, and the maximum was 200 mmHg, with a mean of 141.753 SD ± 21.907 mmHg. The minimum diastolic blood pressure found within the cohort was 50 mmHg, the maximum was 120 mmHg, and, according to our calculations, the mean value was 82.01SD ± 12.99 mmHg. The mean heart rate value was 83.309 SD ± 13.329. As concerns the myocardial infarction area, the prevalence was found to be the following: anterior = lateral > inferior, posterior (Table 1).

As shown in the table below (Table 2), we found a significant association between LRR and myocardial damage (peak high-sensitive troponin I), the severity of heart failure post-myocardial infarction (Killip class III/IV), the necessity of inotropes and vasopressors, in-hospital mortality, TIMI score, and kidney dysfunction. 

### 3.1. LRR and Its Predictive Value for Killip Class III, IV

Within our study, a higher LRR value was associated with a higher risk of Killip class III and IV. Through the logistic regression model, as shown in the table below (Table 3), we have created a risk score—the linear combination of the coefficients and the corresponding predictor values. The risk score of a patient developing Killip class III and IV = intercept coefficient + (LRR coefficient × LRR value). Let us assume that the LRR value is 0.0055 (a value above the cut-off provided by the ROC curve in the figure below). In this case, the risk score of developing Killip class III and IV = −3.5675 + (698.0142 × 0.0055) = 0.271. By using the resulting score, we can calculate the probability of Killip class III and IV occurrence using the formula: probability = 1/(1 + e^score^. Thus, the probability that a STEMI patient with an LRR value of 0.055 will have an evolution consistent with Killip class III and IV is 56.7%.

**Figure 1:**
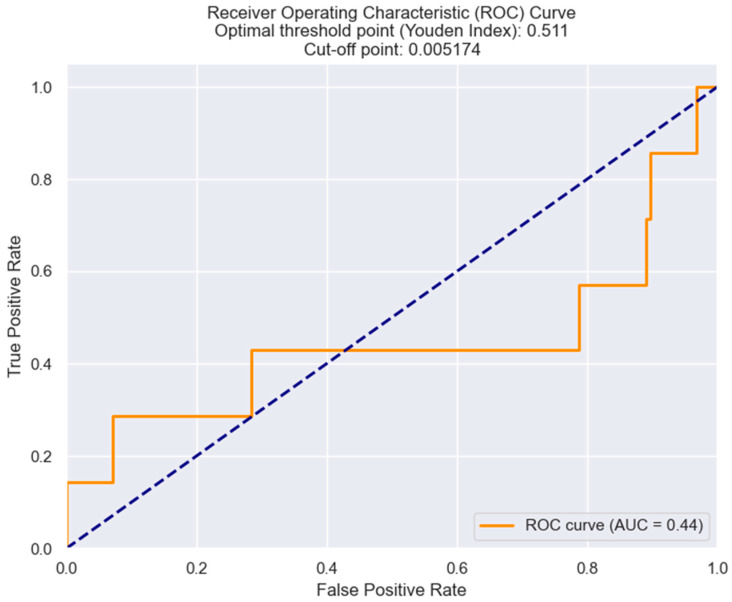
ROC curve analysis for predicting Killip class III/IV based on LRR values in STEMI individuals. The figure is an original contribution by the authors.

The optimal cut-off value of LRR was established through the Youden index from the ROC curve. The resulting cut-off value of LRR for predicting Killip class III and IV in STEMI patients was 0.0051 (Figure 1).

**Figure 2:**
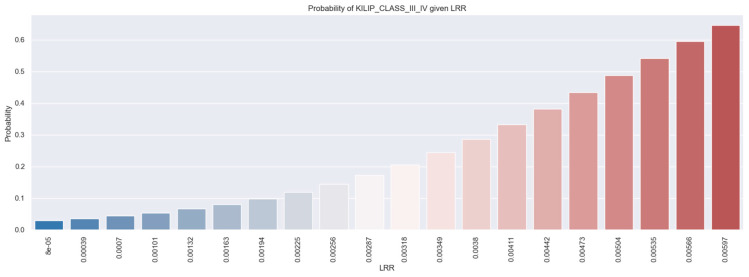
The probability of Killip class III/IV occurrence in STEMI patients based on LRR values. The figure is an original contribution by the authors.

The probability histogram (Figure 2) demonstrates that as the value of the LRR increases, the likelihood that the patient should be categorized as belonging to Killip class III or IV also increases. For example, a patient with an LRR value of 0.0597 has a 65% probability of evolving with Killip class III and IV.

### 3.2. LRR as an Independent Predictive Marker for the Use of Inotropes and Vasopressors

Within the present study, we found a direct correlation between a STEMI patient’s LRR and their probability of necessitating inotropes and vasopressors. The strength of association of the LRR marker with the necessity of inotropes and vasopressors was assessed by a linear regression model (Table 4). As shown below, the *p*-value of LRR (0.014) is below the conventional threshold of 0.05, indicating that the LRR value is statistically significant in predicting the necessity of inotropes and vasopressors in STEMI patients. The logistic regression includes an intercept coefficient of −3.1922 and a coefficient for LRR of 1054.2305. We concluded that the risk score for predicting the necessity of inotropes and vasopressors would be = intercept coefficient + (LRR coefficient × LRR value) = −3.1922 + (1054.2305 × LRR value). Let us consider the case of a patient with an LRR of 0.0032 (this number was not chosen randomly but represents a value above the cut-off established by the ROC in the figure below). We can determine their risk score, which is 0.18, and subsequently calculate the probability of necessitating inotropes and vasopressors by using the formula: probability = 1/(1 + e^score^). In this case, the probability would be 0.455, indicating a 45.5% chance that this patient would need inotropes and vasopressors.

**Figure 3:**
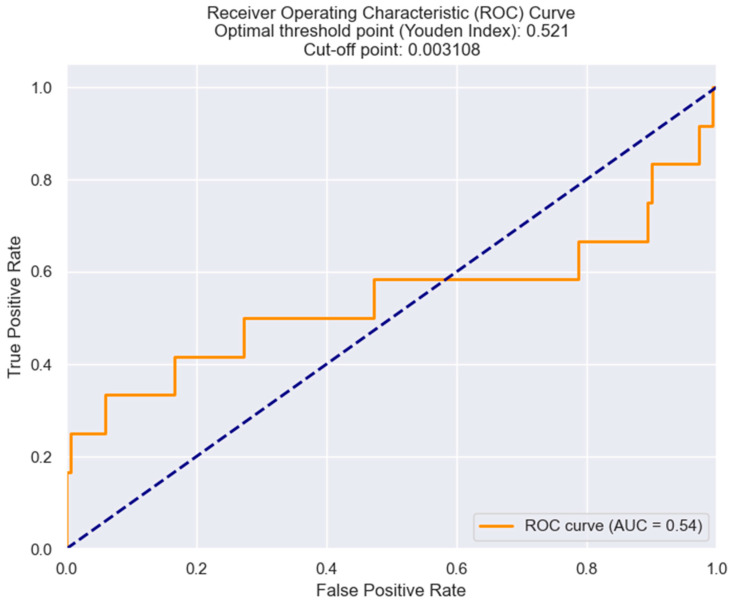
ROC curve analysis for predicting the necessity of inotropes and vasopressors based on LRR values in STEMI individuals. The figure is an original contribution by the authors.

The diagnostic accuracy of the LRR marker for predicting the necessity of inotropes and vasopressors was investigated by means of ROC analysis. The most sensible and specific cut-off value of LRR was found to be 0.0031 (Figure 3).

**Figure 4:**
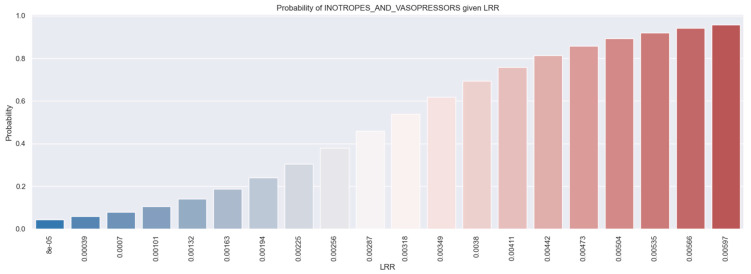
Likelihood of individuals necessitating inotropes and vasopressors derived from LRR values. The figure is an original contribution by the authors.

Furthermore, the probability histogram demonstrates the direct correlation between the LRR value and the necessity of inotropes and vasopressors in STEMI patients (Figure 4). For instance, a patient with an LRR value of 0.00597 has a 66% probability of necessitating inotropic and vasopressor treatment.

### 3.3. LRR—Myocardial Damage and Left Ventricular Stretch

Within our study, we did not find a statistically relevant relationship between the value of LRR and left ventricular stretch (measured by NT proBNP) or LVEF. We also did not demonstrate a statistically significant connection between LRR and admission CK, CK-MB, high-sensitive troponin, or peak CK and CK-MB (Table 5). However, we found a statistically relevant correlation between the value of LRR, and myocardial infarction size represented by peak high-sensitive troponin. Our results reveal that an increase in LRR values correlates with a rise in peak high-sensitive troponin levels. This connection was validated using a linear regression model, with detailed LRR coefficients presented in the accompanying table (Table 5). 

Key findings derived from the data analysis are outlined below.

**Figure 5:**
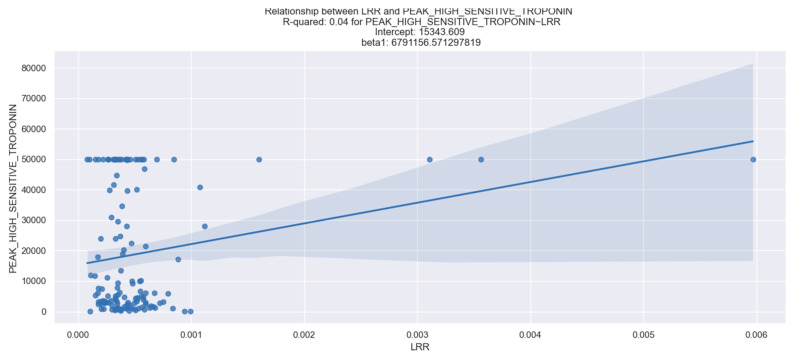
Correlation between LRR and peak high-sensitive troponin. The figure is an original contribution by the authors.

1. The R-squared value of 0.04 indicates that only 4% of the variability in high-sensitive troponin levels can be explained by changes in LRR.
2. The beta coefficient of 6791 suggests that for each 0.001 unit increase in LRR, there is an estimated 0.679 unit increase in high-sensitive troponin levels.
3. The *p*-value for LRR is below 0.05, implying that LRR is a statistically significant predictor of myocardial infarction size. Overall, the data support a positive association between LRR and high-sensitive troponin levels, indicating that higher LRR values are linked to elevated high-sensitive troponin levels, potentially signaling a larger myocardial infarction size. It is important to note, however, that this correlation is weak and explains only a small portion of the variation in high-sensitive troponin levels (Figure 5). We suggest that increasing the sample size could enhance the robustness of this relationship.

### 3.4. LRR and Its Predictive Value for Kidney Dysfunction

**Figure 6:**
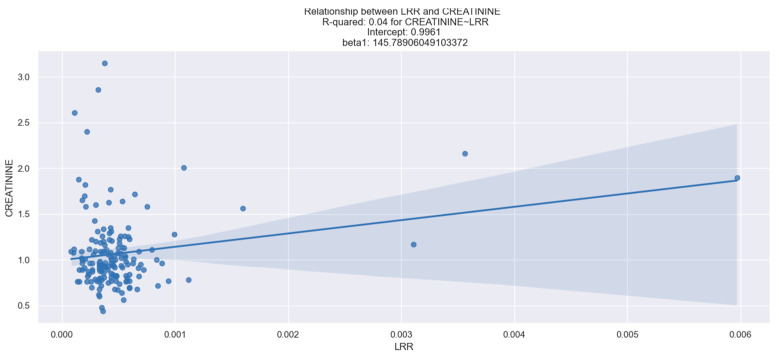
Linear relationship between LRR and creatinine levels. The figure is an original contribution by the authors.

In our study, we observed a reliable relationship between LRR and creatinine levels. Through our analysis, we found that an increase in LRR value was linked to a rise in creatinine levels. This relationship was confirmed through the linear regression model, and the specifics of the LRR coefficients can be seen in Table 6. Key insights gleaned from the data in Table 6 and Figure 6 include the following:

1. The R-squared value of 0.04 suggests that only 4% of the variation in creatinine levels can be explained by the LRR value.
2. The beta coefficient of 145.7891 suggests that for every 0.001 unit increase in LRR, the predicted creatinine level increases by approximately 0.145 units.
3. The *p*-value for LRR is less than 0.05, indicating that LRR is a statistically significant predictor of kidney dysfunction. Overall, the data suggest that there is a positive relationship between LRR and creatinine levels, indicating that an increase in LRR is associated with higher creatinine levels, which may be indicative of kidney dysfunction. However, it is important to note that the relationship is weak and only explains a small portion of the variation in creatinine levels. We believe that a larger sample size can enhance the strength of this relationship.

### 3.5. LRR as an Independent Predictor for In-Hospital Mortality

**Figure 7:**
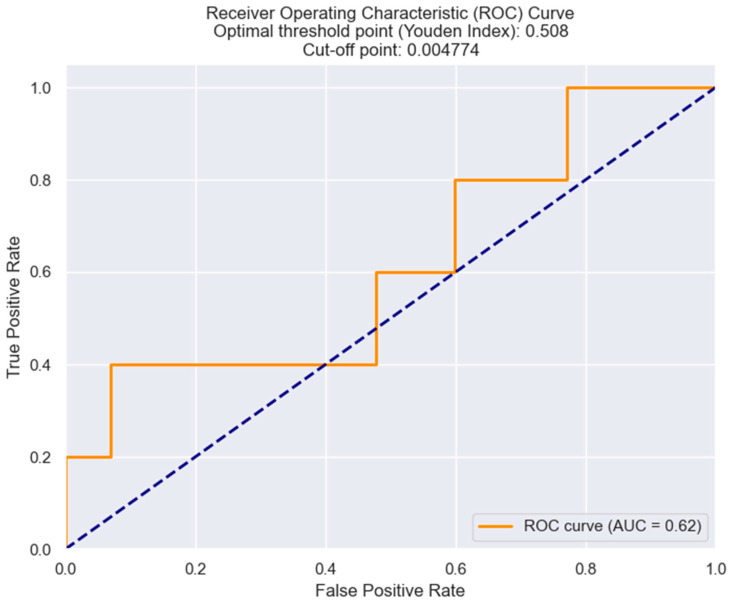
ROC curve analysis for predicting in-hospital mortality derived from LRR values in STEMI individuals. The figure is an original contribution by the authors.

The logistic regression model suggests that the lymphocyte-to-red blood cell ratio (LRR) is a significant predictor for in-hospital mortality in STEMI patients. As illustrated, the model’s pseudo-R-squared value is 0.1264, indicating that the model explains 12.64% of the variance in the dependent variable (in-hospital mortality). The *p*-value of 0.015 is below the conventional threshold of 0.05, indicating that the LRR value is statistically significant in predicting in-hospital mortality. Hence, we are able to calculate a risk score for the probability of in-hospital mortality based on the value of LRR. This risk score = intercept coefficient + (LRR coefficient × LRR value) = −4.1022 + (865.9245 × LRR value). Let us take the example of a patient with an LRR value above the cut-off established through the ROC curve (Figure 7). If the LRR value is 0.005, the score would be = −4.1022 + (865.9245 × 0.005) = 0.227. Using the score, we can determine the probability of in-hospital mortality occurrence using the following formula: probability = 1/(1 + e^score^). In this case, the probability would be 0.577, which signifies that, for this particular patient, there is a 57.7% chance of in-hospital mortality (Table 7).

To determine the optimal cut-off value of LRR for the prediction of in-hospital mortality in STEMI, we utilized the Youden index from the ROC curve. The analysis revealed that an LRR value of 0.0047 was the best threshold, providing optimal sensitivity and specificity (Figure 7).

**Figure 8:**
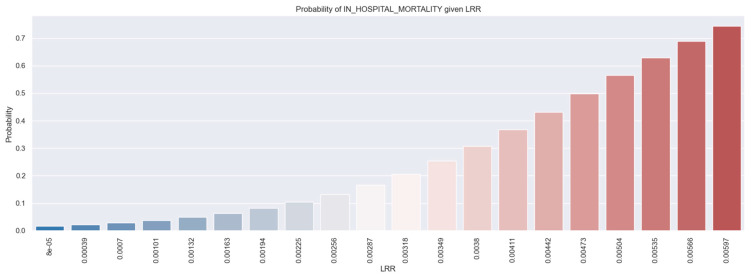
The probability of in-hospital mortality in STEMI individuals based on LRR values. The figure is an original contribution by the authors.

Figure 8 illustrates the relationship between LRR and the probability of in-hospital mortality in STEMI patients. As the value of LRR increases, so does the likelihood of in-hospital mortality. For example, for a patient with an LRR value of 0.00566, there is a 68% probability of dying during their hospitalization.

### 3.6. LRR and TIMI, GRACE and HEART Score

Regarding the connection between LRR and TIMI score in individuals with STEMI, our study utilized a linear regression model to examine the estimated coefficients displayed in Table 8. The findings indicate that there was a relevant relationship between LRR and TIMI score. The higher the LRR value, the higher the TIMI score recorded. This observation aligns with the commonly reported results in the medical literature regarding the significance of the TIMI score in predicting the prognosis of STEMI individuals.

**Figure 9:**
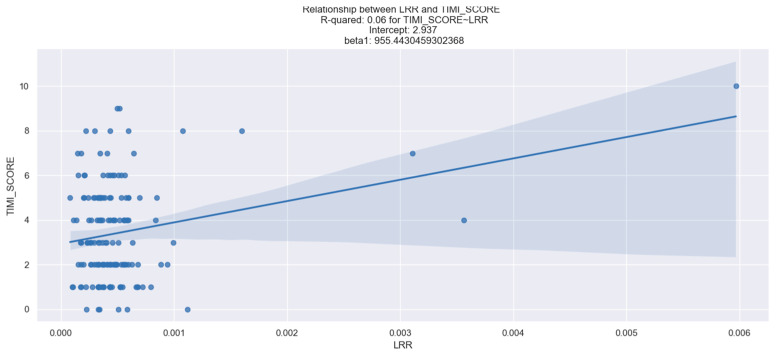
Linear relationship between LRR values and TIMI score. The figure is an original contribution by the authors.

It is important to note, however, that this correlation is weak and explains only a small portion of the variation in TIMI score (Figure 9). We suggest that increasing the sample size could enhance the robustness of this relationship. 

Moreover, we endeavored to determine a correlation between LRR and the GRACE and HEART scores; however, as illustrated in Table 9, no significant connection was demonstrated.

## 4. Discussion

To the best of our knowledge, this study represents the first attempt to investigate the prognostic value of LRR in STEMI patients. Our findings demonstrate that this ratio can serve as a predictive biomarker for assessing the extent of myocardial damage, severity of post-MI heart failure, need for inotropes and vasopressors, high TIMI score, in-hospital mortality, and kidney dysfunction. The predictive values for these primary outcomes were established using ROC curves. Our results indicate that the optimal cut-off point for predicting Killip class III/IV was 0.0051, for the necessity of inotropes and vasopressors was 0.0031, and for in-hospital cardiac mortality was 0.0047. 

Acute ST-segment elevation myocardial infarction continues to be associated with high morbidity and mortality rates. The rapid identification of the high-risk population for acute myocardial infarction mortality remains a significant challenge in current research. Utilizing biomarkers can be pivotal for early prognostic and implementing advanced therapeutic strategies, as they enable the stratification of patients based on personalized risk assessments.

The assessment of acute coronary syndrome patients commonly involves the utilization of the Thrombolysis in Myocardial Infarction risk score-TIMI (history, ECG, age, risk factors, and troponin), HEART, and Global Registry in Acute Coronary Events (GRACE) scoring systems. Despite their widespread use, the ability of these methods to predict major adverse cardiovascular events (MACEs) remains unclear [21,22,23,24,25]. The HEART score, developed in 2008, aims to enhance the precision of diagnosing acute coronary syndrome (ACS) in individuals experiencing undifferentiated chest pain [26]. Similarly, the GRACE score, developed in 2001, is intended for adult patients displaying symptoms of ACS and incorporates various factors such as age, vital signs, kidney function, ECG, and troponin levels [27,28].

To enhance outcomes and develop strategies based on individual risk profiles in STEMI patients, we believe it is necessary to identify new predictive markers. Current clinical risk scores (such as GRACE, TIMI, and HEART) overlook a pivotal predictive element in STEMI: the inflammatory component. Previous studies have highlighted various inflammatory mediators that play crucial roles in acute coronary syndrome [29,30].

Our research has revealed a significant correlation between a high lymphocyte-to-red blood cell ratio (LRR) value and an increased risk of developing Killip class III/IV. Past studies have also suggested that a high lymphocyte count is associated with a poor prognosis [31].

Conversely, some studies propose that a low lymphocyte count is linked to a more severe prognosis in acute coronary syndrome. The underlying reasons for these conflicting results are not entirely clear. One possible explanation for these inconsistencies lies in the differing pro- or anti-atherogenic and inflammatory properties of T and B cell subsets. Given the complex nature of the immune response, the predictive value of these specific subtypes may be more significant than the overall lymphocyte count. Multiple studies have indicated a relationship between an increase in pro-atherogenic CD4 CD28null T cells and a higher incidence of recurring cardiovascular events. This finding could help explain our results, supporting the concept that the activation of these specific T cell subsets contributes to an adverse prognosis in coronary artery disease [32,33]. Our findings are reinforced by literature that has identified a correlation between lymphocyte count and heart failure in ACS patients, with the baseline lymphocyte count recognized as an independent predictor of heart failure in individuals undergoing percutaneous coronary intervention (PCI). Patients with congestive heart failure exhibit elevated pro-inflammatory CD4+ Th1 and Th17 cells alongside reduced anti-inflammatory T regulatory cells (Treg), correlating with disease severity [34].

In relation to myocardial damage, we have demonstrated a statistically significant association between LRR and peak high-sensitive troponin I levels. In acute coronary syndrome, there is a systemic increase in CD4+ and CD8+ T lymphocytes, along with CD4+ Th1, Th2, Th17, and Treg subsets in cardiac, circulatory, and lymphoid tissues. Studies have underscored the critical role of T lymphocytes in cardiac remodeling post-myocardial infarction (MI) [35]. Our findings align with previous studies suggesting that CD4/CD28-null T cells tend to accumulate in unstable ruptured coronary plaques, contributing to plaque instability [36].

Additionally, our investigation revealed a statistically significant correlation between high LRR levels and kidney dysfunction in STEMI patients. Upon reviewing the literature, the etiology of renal injury in ACS patients is intricate, arising from a combination of factors such as hemodynamic abnormalities due to reduced cardiac output and venous return, activation of the sympathetic nervous system, and the renin–angiotensin–aldosterone system (RAAS), leading to vasoconstriction and exacerbating kidney damage, disruptions in the coagulation system, activation of the inflammatory response, and oxidative stress [37,38,39,40]. In line with our results, research has highlighted the substantial involvement of lymphocytes in acute kidney injury (AKI). Th1 cells release pro-inflammatory cytokines such as interferon-gamma (IF-) and interleukin-2 (IL-2), while Th2 cells predominantly secrete IL-4 and IL-10 (recognized for their anti-inflammatory properties) among other cytokines. Both types of cells are believed to contribute to the initial injury phase of ischemia–reperfusion [41,42].

Studies have indicated that elevated levels of IL-10 are linked to adverse outcomes in ACS and the development of AKI post-cardiac surgery. Similarly, heightened IL-10 levels in cases of renal dysfunction are predictive of mortality in acute circumstances [43,44,45].

Exploring the pathophysiological mechanisms further, we discover more insights into our findings, particularly concerning the relationship between erythrocytes and lymphocytes. The interaction between red blood cells and leukocytes has predominantly been studied in patients with neoplasia. Several recent studies have suggested that red blood cells have the potential to modulate T cell proliferation [46,47]. During states of high oxidative stress, oxidized red blood cells release hemoglobin, heme-Fe, and iron, potent sources of oxidation and radicals. These molecules can activate endothelial cells and innate immune cells in a pro-inflammatory manner [48]. Evidence indicates that red blood cells may influence T cell proliferation, both in vivo and in vitro. Amid the complex pathophysiology of myocardial infarction, the interplay between red blood cells and other hematological cells may offer predictive value. However, the studies focusing on atherosclerosis processes and high oxidative stress primarily target carotid atherosclerosis, overlooking myocardial infarction [15,49]. With this insight as a foundation, we conducted a study on a single-center cohort of STEMI patients to explore the potential of LRR in predicting prognosis. Moreover, LRR is an independent predictor of short-term mortality, Killip class III/V, size of myocardial infarction, necessity of inotropes and vasopressors, and kidney dysfunction. Despite this, we were unable to establish a link between the GRACE and HEART scores and LRR. Our newly suggested predictive marker shows correlations with heart failure post-ACS and renal dysfunction, outcomes that are part of the GRACE score. This marker not only forecasts in-hospital mortality but also predicts the likelihood of necessitating inotropes and vasopressors and the extent of myocardial damage—all of which are not covered by the GRACE or HEART score. This predictive marker we introduce acts as proxies for the underlying pathophysiological processes during ACS, going beyond what the GRACE and HEART score consider clinically and biologically. This could explain the lack of correlation between LRR and the GRACE and HEART score. Furthermore, the lymphocyte to RBC ratio may not have been correlated to GRACE and HEART scores because these tools predict mortality not only in the inpatient phase, but also in the longer run.

Larger studies with multicenter patient cohorts are necessary to confirm or refute this lack of correlation, given the relatively small sample size in our study.

Our findings underscore the immense potential of this simple algorithm in providing crucial short-term risk assessment for STEMI patients, even prior to revascularization. The LRR serves as a comprehensive reflection of the intricate interplay between lymphocytes and red blood cells amidst acute coronary syndrome. Our research unveils the profound impact of erythrocytes navigating high oxidative stress environments, undergoing transformative changes while emerging as potent sources of oxidation and radicals, exacerbating the inflammatory environment and influencing T cell dynamics. Importantly, our results shed light on the correlation between elevated LRR levels, intensified inflammatory responses, and dismal prognoses. Previous studies have highlighted the detrimental implications of diminished lymphocyte counts in acute coronary syndrome, attributed to hormonal and sympathetic nervous system dysregulation, albeit overlooking the synergistic relationship between erythrocytes and lymphocytes. The LRR emerges as a cost-effective, reliable, and easily accessible inflammatory biomarker with significant implications for risk stratification in STEMI patients, offering clinicians an invaluable tool to enhance outcome predictions and mitigate adverse events in high-risk cohorts.

### 4.1. Limitations

In our research, we encountered several limitations. Initially, we only evaluated the lymphocyte/red blood cell ratio of each patient upon admission, without subsequent assessments. An optimal strategy would involve measuring these parameters at various stages and conducting comparative investigations. Additionally, this study was confined to a single center with a relatively limited patient cohort, emphasizing the need for future extensive, cross-center studies. Furthermore, we failed to follow the long-term prognosis of these individuals, which can limit the predictive value of LRR for long-term risk.

### 4.2. Future Directions

The undeniable connection between LRR and STEMI prognosis highlights the importance of delving deeper into the pathophysiology of STEMI in the early stages of the process. Understanding the function of blood cells is crucial for enhancing prognostic evaluation, refining therapeutic strategies, and better risk categorization. Continued investigation has the potential to pave the way for tailored and effective treatment plans for individuals with STEMI.

### 4.3. Ethical Approval and Informed Consent

The study was conducted according to the guidelines of the Declaration of Helsinki and approved by the Ethics Committee of “Carol Davila” University of Medicine and Pharmacy, Bucharest, Romania (protocol code PO-35-F-03, date 1 October 2021). Informed consent was obtained from all subjects involved in this study.

## 5. Conclusions

In the present study, we have effectively demonstrated the lymphocyte-to-red blood cell ratio (LRR) as an independent predictive marker for STEMI complications. Moreover, we have pinpointed crucial LRR threshold values that can accurately distinguish patients at increased risk of complications, thereby enabling improved treatment approaches. 

Due to its simplicity, affordability, accessibility, and cost-effectiveness, LLR can be effectively used to anticipate complications in STEMI patients, helping to determine the most suitable course of management. 

## Figures and Tables

**Table 1:** Clinical, demographical, and main biological characteristics of the study population.

Variable	Mean	STD	MIN	25%	50%	75%	MAX
Age	63.423	10.418	41	56	64	72	89
Female	0.361	0.483	0	0	0	1	1
Male	0.639	0.483	0	0	1	1	1
Smoker	0.515	0.502	0	0	1	1	1
BMI > 30	0.361	0.483	0	0	0	1	1
Arterial hypertension	0.794	0.407	0	1	1	1	1
Diabetes mellitus	0.392	0.491	0	0	0	1	1
Dislipidemia	0.567	0.498	0	0	1	1	1
(63.9%) Old myocardial infarction	0.237	0.428	0	0	0	0	1
Systolic blood pressure	141.753	21.907	90	130	140	155	200
Diastolic blood pressure	82.01	12.99	50	74	80	90	120
Heart rate	83.309	13.329	55	75	80	90	120
Anterior STEMI	0.031	0.175	0	0	0	0	1
Inferior STEMI	0.021	0.144	0	0	0	0	1
Lateral STEMI	0.031	0.175	0	0	0	0	1
Right ventricle STEMI	0	0	0	0	0	0	0
Posterior STEMI	0.021	0.144	0	0	0	0	1

The table is an original contribution by the authors.

**Table 2:** Correlation between LRR and outcomes in STEMI patients.

Dependent Variable	r Squared	Independent Variable	Coefficient	*p* Value
Admission CK (U/L)	0.000	LRR	10,880	0.916
Admission CK-MB (U/L)	0.004	LRR	43,530	0.34
Peak CK (U/L)	0.005	LRR	132,400.0	0.376
Peak CK-MB (U/L)	0.000	LRR	6421.1859	0.82
Admission-high-sensitive troponin I (ng/mL)	0.000	LRR	134,300.0	0.943
Peak high-sensitive troponin I (ng/mL)	0.042	LRR	6,791,000.0	0.015
Inotropes and vasopressors	0.1052	LRR	1054.2305	0.014
Killip class III/IV	0.06794	LRR	698.0142	0.033
In-hospital mortality	0.1264	LRR	865.9245	0.015
NT-proBNP (pg/mL)	0.010	LRR	731,200.0	0.342
LVEF (%)	0.002	LRR	−840.7903	0.567
HEART Score	0.007	LRR	198.1893	0.308
GRACE Score	0.006	LRR	3788.5318	0.322
TIMI Score	0.065	LRR	955.443	0.001
Creatinine (mg/dL)	0.044	LRR	145.7891	0.008
30-days mortality	0.01205	LRR	−1556.7879	0.594

The table is an original contribution by the authors.

**Table 3:** Logistic regression analysis for the presence of Killip III/IV based on LRR value.

Dep. Variable	Killip Class III/IV	No. Observations	162			
	Coefficient	Std err	Z	P > |z|	0.025	0.975
Intercept	−3.5675	0.482	−7.396	0.0	−4.513	−2.622
LRR	698.0142	326.958	2.135	0.033	57.188	1338.841

The table is an original contribution by the authors.

**Table 4:** Logistic regression analysis for the necessity of inotropes and vasopressors based on LRR value.

Dep. Variable:	Inotropes and Vasopressors	No. Observations:	162			
	Coefficient	std err	Z	P > |z|	0.025	0.975
Intercept	−3.1922	0.421	−7.574	0.0	−4.018	−2.366
LRR	1054.2305	427.424	2.466	0.014	216.494	1891.967

The table is an original contribution by the authors.

**Table 5:** Logistic regression analysis for high-sensitive troponin levels based on LRR value.

Dep. Variable:	High-Sensitive Troponin	No. Observations:	162			
	Coef	std err	Z	P > |z|	0.025	0.975
Intercept	15,340	2191.977	7.0	0.0	11,000	19,700
LRR	6791	2740000	2.476	0.015	1,370,000	12,200,000

The table is an original contribution by the authors.

**Table 6:** Logistic regression analysis for creatinine levels based on LRR value.

Dep. Variable:	Creatinine	No. Observations:	162			
	Coef	Std err	Z	P > |z|	0.025	0.975
Intercept	0.9961	0.041	24.137	0.0	0.915	1.078
LRR	1,457,891	54.032	2.698	0.008	39.081	252.497

The table is an original contribution by the authors.

**Table 7:** Logistic regression analysis for predicting in-hospital mortality derived from LRR value.

Dep. Variable	Intrahospital Mortality	No. Observations	162			
	Coef	Std err	Z	P > |z|	0.025	0.975
Intercept	−2.9188	0.377	−6.915	0.0	−5.265	−2.939
LRR	698.7193	319.208	2.437	0.015	169.644	1562.205

The table is an original contribution by the authors.

**Table 8:** Logistic regression analysis for the correlation LRR-TIMI score.

Dep. Variable	TIMI Score	No. Observations	158			
	Coefficient	Std err	Z	P > |z|	0.025	0.975
Intercept	2.937	0.224	13.133	0.0	−2.495	3.379
LRR	955.443	290.655	3.287	0.001	381.317	1529.57

The table is an original contribution by the authors.

**Table 9:** Association between LRR and GRACE and HEART score.

Dependent Variable	r Squared	Independent Variable	Coefficient	*p*-Value
GRACE Score	0.006	LRR	3788.5318	0.322
HEART Score	0.007	LRR	198.1893	0.308

The table is an original contribution by the authors.

## Data Availability

The data presented in this study are available on request from the corresponding author. The data are not publicly available due to privacy issues.
